# Cross-Neutralizing Breadth and Longevity Against SARS-CoV-2 Variants After Infections

**DOI:** 10.3389/fimmu.2022.773652

**Published:** 2022-02-24

**Authors:** Yukiya Kurahashi, Silvia Sutandhio, Koichi Furukawa, Lidya Handayani Tjan, Sachiyo Iwata, Shigeru Sano, Yoshiki Tohma, Hiroyuki Ohkita, Sachiko Nakamura, Mitsuhiro Nishimura, Jun Arii, Tatsunori Kiriu, Masatsugu Yamamoto, Tatsuya Nagano, Yoshihiro Nishimura, Yasuko Mori

**Affiliations:** ^1^ Division of Clinical Virology, Center for Infectious Diseases, Kobe University Graduate School of Medicine, Kobe, Japan; ^2^ Division of Cardiovascular Medicine, Hyogo Prefectural Kakogawa Medical Center, Kakogawa, Japan; ^3^ Acute Care Medical Center, Hyogo Prefectural Kakogawa Medical Center, Kakogawa, Japan; ^4^ Division of General Internal Medicine, Hyogo Prefectural Kakogawa Medical Center, Kakogawa, Japan; ^5^ Division of Respiratory Medicine, Hyogo Prefectural Awaji Medical Center, Sumoto, Japan; ^6^ Division of Respiratory Medicine, Department of Internal Medicine, Kobe University Graduate School of Medicine, Kobe, Japan

**Keywords:** SARS-CoV-2, COVID-19, variants, neutralization, breadth

## Abstract

**Background:**

Severe Acute Respiratory Syndrome Coronavirus 2 (SARS-CoV-2) is the virus responsible for the Coronavirus Disease 2019 (COVID-19) pandemic. The emergence of variants of concern (VOCs) has become one of the most pressing issues in public health. To control VOCs, it is important to know which COVID-19 convalescent sera have cross-neutralizing activity against VOCs and how long the sera maintain this protective activity.

**Methods:**

Sera of patients infected with SARS-CoV-2 from March 2020 to January 2021 and admitted to Hyogo Prefectural Kakogawa Medical Center were selected. Blood was drawn from patients at 1-3, 3-6, and 6-8 months post onset. Then, a virus neutralization assay against SARS-CoV-2 variants (D614G mutation as conventional strain; B.1.1.7, P.1, and B.1.351 as VOCs) was performed using authentic viruses.

**Results:**

We assessed 97 sera from 42 patients. Sera from 28 patients showed neutralizing activity that was sustained for 3-8 months post onset. The neutralizing antibody titer against D614G significantly decreased in sera of 6-8 months post onset compared to those of 1-3 months post onset. However, the neutralizing antibody titers against the three VOCs were not significantly different among 1-3, 3-6, and 6-8 months post onset.

**Discussion:**

Our results indicate that neutralizing antibodies that recognize the common epitope for several variants may be maintained for a long time, while neutralizing antibodies having specific epitopes for a variant, produced in large quantities immediately after infection, may decrease quite rapidly.

## Introduction

Severe Acute Respiratory Syndrome Coronavirus 2 (SARS-CoV-2) is the virus responsible for the Coronavirus Disease 2019 (COVID-19) pandemic, which began in November, 2019. Most COVID-19 cases are asymptomatic to mild, but some cases lead to life-threatening pneumonia. As of mid-December, 2021, more than 5.4 million patients worldwide have died from the effects of COVID-19 (1).

To control this pandemic, various prophylactic and therapeutic approaches are being tried clinically, including vaccines, convalescent plasma therapy (CPT) and therapeutic monoclonal antibodies (Mabs) (2). Among these immunotherapies, the neutralizing antibodies (Nabs) that interrupt viral infection are essential components and are induced by natural infection or vaccination. Some SARS-CoV-2 vaccines, including messenger RNA-based vaccines and adenovirus-vectored vaccines, have a high efficacy at preventing symptomatic disease (3). Numerous different vaccines have been manufactured and distributed all over the world, but the production has not been sufficient to vaccinate populations. The rapid increase of vaccine supply remains the best hope for overcoming this pandemic.

On the other hand, passive immunization using CPT remains a therapeutic option and has been used for infectious diseases caused by severe acute respiratory syndrome coronavirus (4), Middle East Respiratory Syndrome Coronavirus (4), and Ebola virus (5). The efficacy of CPT for COVID-19 patients is controversial (6), although some studies provided good evidence that CPT was safe and reduced mortality when COVID-19 patients were treated in combination with antiviral drugs, steroids, and other supportive care (7). Treatment with therapeutic Mabs is another option as a passive immunotherapy with no risk of post-transfusion infection and no need to collect convalescent sera from many recovered patients. Mabs are important for immunocompromised people and unvaccinated people to be protected from infection. Currently, Mabs for five diseases (respiratory syncytial virus, anthrax, *Clostridium difficile*, human immunodeficiency virus 1, and Ebola virus) are approved by the US Food and Drug Administration (FDA) (8, 9). As for SARS-CoV-2, three anti-SARS-CoV-2 Mabs products (sotrovimab, combination of bamlanivimab plus etesevimab, and combination of casirivimab plus imdevimab) have Emergency Use Authorizations (EUAs) from the FDA (10). These Mabs products are used for treatment of mild to moderate COVID-19 nonhospitalized patients to prevent hospitalization and death. Combination of casirivimab plus imdevimab has obtained fast-track approval of Japan’s Ministry of Health, Labour, and Welfare on 19 July 2021 (11).

In the face of these new therapies, SARS-CoV-2 has evolved from its original strain. The D614G mutation of spike protein (S protein) was found worldwide by the end of June, 2020 (12). Recently, the emergence of SARS-CoV-2 variants with mutations that can enhance transmissibility and reduce neutralization activity has brought new challenges to the management of COVID-19. The World Health Organization (WHO) classified five variants (B.1.1.7, P.1, B.1.351, B.1.617.2, and B.1.1.529) as variants of concern (VOC) (13). B.1.1.7, which was firstly detected in the United Kingdom, has an N501Y mutation in the receptor binding domain (RBD) of S protein. P.1, which was identified in Brazil, has three mutations (K417T, E484K, and N501Y) in the RBD. B.1.351, which was found in South Africa, has three mutations (K417N, E484K, and N501Y) in the RBD (14). B.1.617.2, which was confirmed in India, has mutations (L452R and T478K) in the RBD (15), leading to higher viral load in infected individuals, in addition to the P681R mutation which increases the virus transmissibility (16). Finally, B.1.1.529, which was detected in Botswana on November 11, 2021 and South Africa on November 14, 2021, has 15 mutations (G339D, S371L, S373P, S375F, K417N, N440K, G446S, S477N, T478K, E484A, Q493R, G496S, Q498R, N501Y, and Y505H) in the RBD (17). The N501Y mutation shared among B.1.1.7, B.1.351, P1 and B.1.1.529 have influence on the affinity between the angiotensin-converting enzyme 2 (ACE2) receptor and the RBD of the S protein, which causes high transmissibility of the virus. The variant B.1.1.7 has proven to be more transmissible than original strains (18). The E484K mutation in the RBD of B.1.351 and P1 is involved in immune escape (19, 20). The K417N mutation of B.1.351 and B.1.1.529 and K417T mutation of P.1 are suggested to change the conformation of S protein, allowing escape from Nabs (19, 20). Nabs have reduced activity on the variants B.1.351 and P.1 (14). Especially, the variant B.1.351 is much more resistant to neutralization by Nabs, possibly because of differences in the mutations of the N terminal domain (NTD), which are associated with escape from immunity (21). Nabs against B.1.1.529 from sera of convalescent patients infected with the ancestral SARS-CoV-2 and sera of vaccinated people with two dose of ChAdOx1 nCoV-19 or BNT162b2 is also lower than that against the other variants (22, 23).

As elsewhere in the world, Japan is facing COVID-19 pandemic. As of mid-December 2021, nearly 1,730,000 Japanese people had been infected with COVID-19 and about 18,000 people had died (1). So far, the country has been affected by five waves of exponential increase in new cases. D614G_KR, which had D614G mutation of S protein and 203_204delinsKR mutation of nucleocapsid protein, and its lineage were predominant during the first to third waves, from March 2020 to February 2021 (24). VOCs have also spread to Japan. B.1.1.7 was first detected on 25 December 2020 and rapid community spread occurred from March 2021. As of June 2021, B.1.1.7 became the predominant variant in Japan. In contrast, B.1.351 and P.1 were not spread widely in Japan, although they were found at the end of December 2020 and the beginning of January 2021. B.1.617.2 was detected on 20 April 2021 and has rapidly replaced B.1.1.7. Then, B.1.617.2 has become the predominant variant at the fifth wave from July 2021 in Japan (25, 26). Recently, B.1.1.529 was detected in Japan as in other countries. Then, the Japanese government is on the alert for the sixth wave (27).

Hence, it is important to know about the longevity of the neutralizing activity of convalescent sera against SARS-CoV-2 to estimate the possibility of reinfection, to select good donors for CPT, and to make therapeutic Mabs products. Previous studies have shown that neutralizing activity of convalescent sera is maintained up to six to twelve months post onset, although follow-up studies for longer duration are still needed (28–30). Furthermore, the breadth and longevity of cross-neutralizing activities against VOCs have been minimally tested (28, 31). In this study, we analyzed the longevity and breadth of neutralizing activity of COVID-19 convalescent sera across the VOCs (B1.1.7, P.1, and B.1.351) and the D614G.

## Methods

### Diagnosis of COVID-19 and Definition of Severities

Polymerase chain reaction (PCR) detection of the SARS-CoV-2 genome in nasopharyngeal swab samples was used to confirm the diagnosis of COVD-19. We used the same definitions of severities as in our previous report (32).

Asymptomatic patients had neither clinical symptoms nor hypoxia. Patients with mild illness had symptoms without evidence of pneumonia or hypoxia. Those with moderate illness had clinical symptoms of pneumonia with oxygen saturation levels over 90% on room air. Those with severe illness suffered from pneumonia with an oxygen saturation level under 90% on room air. Patients who needed mechanical ventilation were classified as critical.

### Study Site and Patient Recruitment

From March 2020, blood samples of COVID-19 patients have been collected by Hyogo Prefectural Kakogawa Medical Center, located at Kakogawa, Hyogo, Japan. In this study, samples of patients infected from March 2020 to January 2021 were selected. Serial blood samples were collected from individuals who had different severities at various time points post onset: 1-3 months post onset, 3-6 months post onset, and 6-8 months post onset.

### Virus Strains

The SARS-CoV-2 Biken-2 (B2) strain including the D614G mutation was used as the conventional virus (accession number: LC644163), and was received from BIKEN Innovative Vaccine Research Alliance Laboratories. The three SARS-CoV-2 variants: B.1.1.7 (GISAID ID: EPI_ISL_804007), P.1 (GISAID ID: EPI_ISL_833366), and B.1.351 (GISAID ID: EPI_ISL_1123289) were received from National Institute of Infectious Diseases (NIID), Tokyo, Japan. Mutations of genes encoding spike protein were confirmed by cDNA sequencing.

### Neutralization Assay

The live virus neutralization assay against SARS-CoV-2 variants (D614G, B.1.1.7, P.1, and B.1.351) was done as previously reported according to Biosafety Level 3 regulations (32, 33). At 24 hours before the assay, 4 × 10^4^ Vero E6 (TMPRSS2) cells per well were seeded in 96-well tissue culture microplates. Serum samples were heat-inactivated at 56°C for 30 minutes, and two-fold serially diluted using Dulbecco’s Modified Eagle’s Medium as the diluent. Diluted serum samples were mixed with 100 tissue culture infectious dose (TCID)_50_ of SARS-CoV-2 variants and incubated at 37°C for one hour. The mixture of sera and virus was added to confluent Vero E6 (TMPRSS2) cells in a 96-well plate. Cells were incubated at 37°C with 5% CO_2_ supplementation for six days. Then, the neutralizing titer was determined as the dilution factor in which cells showed no cytopathic effect. The titer was shown on a log2 scale. The cutoff titer was set at one; titer under one was defined as ND (not detected).

### Statistical Analysis

Continuous variables are described using medians and interquartile ranges (IQRs), defined by the 25th and 75th percentiles. Categorical factors were reported as counts and percentages. Cochran’s Q test and Benjamini–Hochberg correction were performed to compare the proportion of patients whose Nab titer was ND among four variants. Friedman’s test and the Benjamini–Hochberg correction was performed to compare the Nab titers among variants or sampling times. The level of statistical significance was set at *p* < 0.05. Statistical analyses were performed using STATA (version 14.2). Sample size calculation was not performed.

### Ethics

Our study was approved by the ethics committee of Kobe University Graduate School of Medicine (ID: B200200) and Hyogo Prefectural Kakogawa Medical Center. Written consent or the opt-out consent for our observational study was obtained.

## Results

### Characteristics of Patients

We assessed 97 sera from 42 individuals in total, and the data are shown in **Table 1** and **Supplementary Table 1**. The median age with IQR was 56 (49–62) years. Fifty percent of patients were female. Patients’ blood was taken two or three times serially. In terms of disease severity, four patients were asymptomatic (P1 to P4), fifteen had mild disease (P5 to P19), five had moderate disease (P20 to P24), fifteen had severe disease (P25 to P39) and three were critical (P40 to P42). We divided the post-onset data according to the three time periods (1-3 months, 3-6 months, and 6-8 months). The median days with IQR for these assessments were 47 (43–54), 117 (110–132), and 209 (199–219). Common chronic conditions were hypertension (28.6%), diabetes (26.2%), and pulmonary diseases (19.0%) including asthma and chronic obstructive pulmonary disease (COPD).

**Table 1:** Characteristics of patients.

Characteristics of patients (N = 42)			
			Total (N = 42)
Age, y, median (IQR)			56 (49–62)
Sex (Female), n (%)			21 (50)
Serial sampling, n (%)	twice		29 (69.0)
	three times		13 (31.0)
Months post onset n (%)	1-3 months		38 (90.5)
	3-6 months		42 (100)
	6-8 months		17 (40.5)
Severity, n (%)	pneumonia (–)	asymptomatic	4 (9.5)
		mild	15 (35.7)
	pneumonia (+)	moderate	5 (11.9)
		severe	15 (35.7)
		critical	3 (7.1)
Therapy, n (%)	steroid		20 (47.6)
	antiviral drug		4 (9.5)
Past medical history, n (%)	hypertension		12 (28.6)
	cardiovascular disease		2 (4.8)
	pulmonary disease		8 (19.0)
	diabetes		11 (26.2)
	chronic kidney disease		1 (2.4)
	cancer		1 (2.4)

### Longevity of Neutralizing Activity Against D614G and VOCs

**Figure 1:**
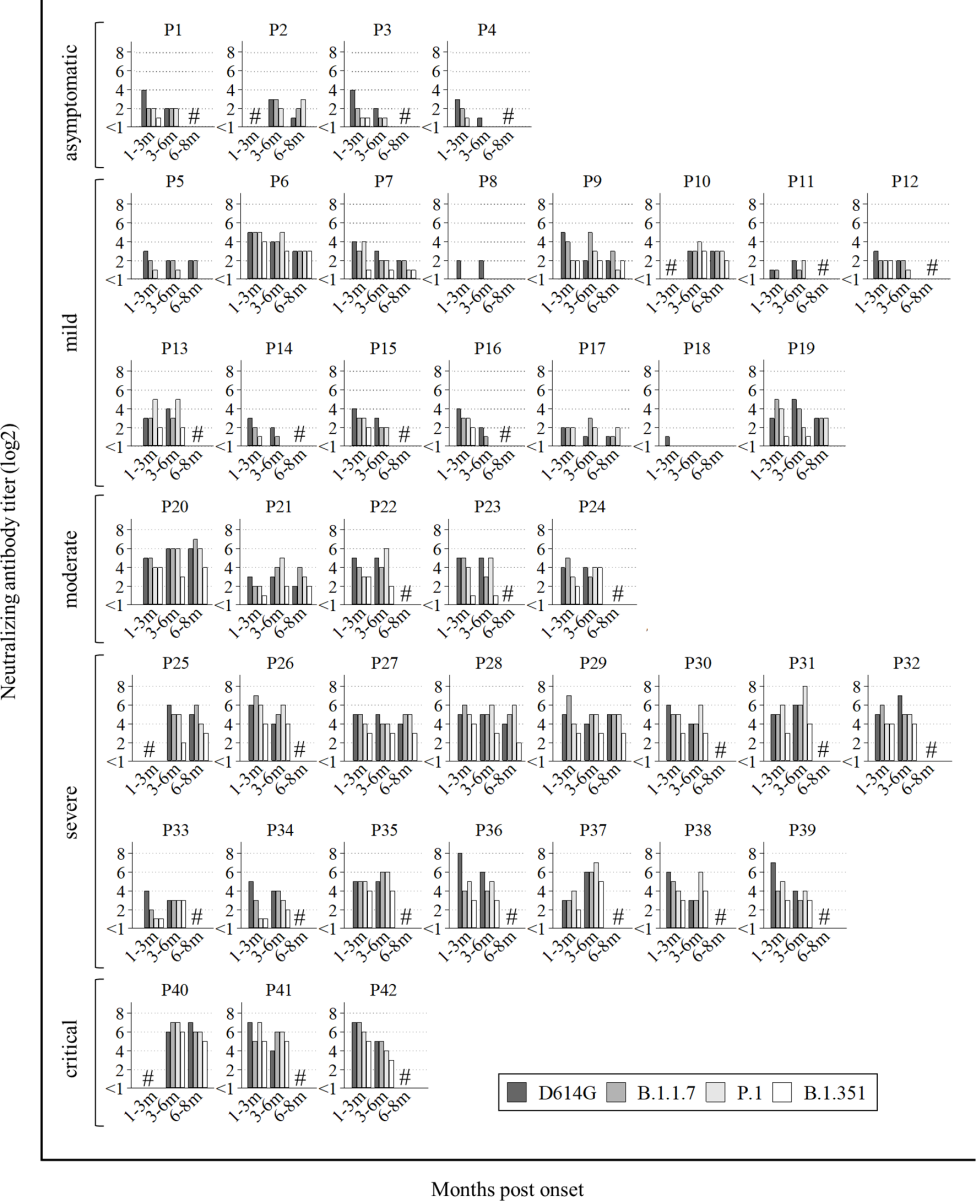
All data of neutralization assay. P1 to P4 were asymptomatic, P5 to P19 had mild disease, P20 to P24 had moderate disease, numbers P25 to P39 had severe disease and P40 to P42 were critical. Hash marks (#) on the graphs means no sera at the points. Vertical bars show the neutralizing antibody titer (log2). Horizontal bars show the trend among patients at 1-3 months post onset, 3-6 months post onset, and 6-8 months post onset according to the four variants (D614G mutation, B.1.1.7, P.1, and B.1.351).

All data of neutralization assays are shown in **Figure 1**. Among 42 patients, sera from 28 patients showed long-lasting neutralizing activities on the three VOCs (five out of fifteen mild: P6, P7, P9, P10, and P13, and all moderate to critical), in addition to D614G. On the other hand, sera from two patients (P8 and P18) showed no cross-neutralizing activity for any VOCs, and sera from six patients (P2, P4, P5, P11, P14, and P17) did not neutralize B.1.351 at all. Sera from the other six patients (P1, P3, P12, P15, P16, and P19) neutralized B.1.351 at first, but later could not. Totally, all four asymptomatic and 10 out of 15 mild patients could not obtain or maintain cross-Nabs for the three variants.

**Figure 2:**
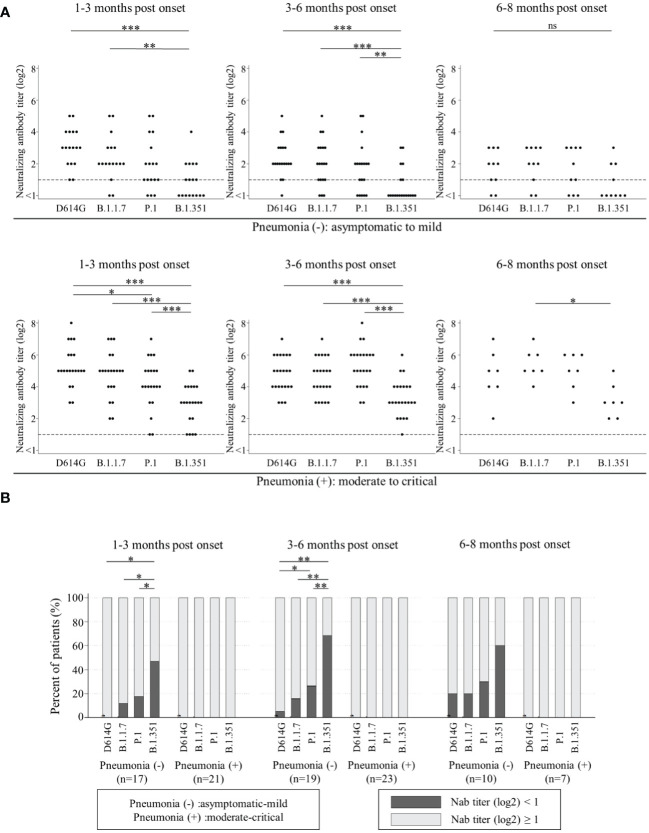
Comparisons of neutralizing antibody titers among D614G and three VOCs by the timing of sampling in ‘patients without pneumonia’ [1-3 months post onset (n=17), 3-6 months post onset (n=19), and 6-8 months post onset (n=10)] and those in ‘patients with pneumonia’ [1-3 months post onset (n=21), 3-6 months post onset (n=23), and 6-8 months post onset (n=7)] are shown in **(A)**. Vertical bars show the neutralizing antibody titer (log2). Horizontal bars show the four variants (D614G mutation, B.1.1.7, P.1, and B.1.351) by the three groups of months post-onset (1-3m, 3-6m, and 6-8m). Friedman’s test and Benjamini–Hochberg correction were performed to analyze both data. The dash line shows that neutralizing antibody titers (log2) is one, which is the cut-off point. Comparison of neutralizing titer under one (ND) among four variants are shown in **(B)**. Vertical bars show the percentages of patients. Black shows the percentage of neutralizing antibody titers (log2) under one and grey shows that of more than one. Horizontal bars show the four variants (D614G mutation, B.1.1.7, P.1, and B.1.351) by the three sampling times. Cochran’s Q test and Benjamini–Hochberg correction were performed. The level of statistical significance was set at *p* < 0.05 (*p < 0.05; **p < 0.01; ***p < 0.001; and ns, not significant)).

Next, we analyzed the Nab titers among D614G and three VOCs by two severity groups. One group was named ‘patients without pneumonia’. This group included the patients who did not present with pneumonia (asymptomatic and mild patients). The other was named ‘patients with pneumonia’, including the patients who presented with pneumonia (moderate, severe and critical patients). The Nab titers against B.1.351 in both groups were significantly lower than those against the other variants at 1-3 months post onset and 3-6 months post onset. The Nab titers against all four variants in ‘patients with pneumonia’ were higher than those in ‘patients without pneumonia’ (**Figure 2A**).

Then, we analyzed the trend of ND (not detected, that is, Nab titer under one) among D614G and three VOCs by two severity groups in **Figure 2B**. All in ‘patients with pneumonia’ acquired and maintained cross-Nabs for three VOCs. However, many patients in ‘patients without pneumonia’ could not acquire or maintain the Nab titers for D614G and three VOCs. The proportions of patients with ND for D614G, B.1.1.7, and P.1 at 1-3 months post onset were 0%, 11.8%, and 17.6%, respectively. The proportion of patients with ND for B.1.351 was 47.1% and significantly higher than the other variants. The proportion of patients with ND for P.1 was the second-highest but without significant difference from D614G and B.1.1.7. The proportions of patients with ND for D614G, B.1.1.7, P.1 and B.1.351 at 3-6 months post onset were 5.3%, 15.8%, 26.3%, and 68.4%, respectively. The difference among four variants at 3-6 months post onset was similar to that at 1-3 months post onset. The proportions of patients with ND for D614G, B.1.1.7, P.1, and B.1.351 at 6-8 months post onset were 20%, 20%, 30%, and 60%, respectively. There was no significant difference at this time point.

**Figure 3:**
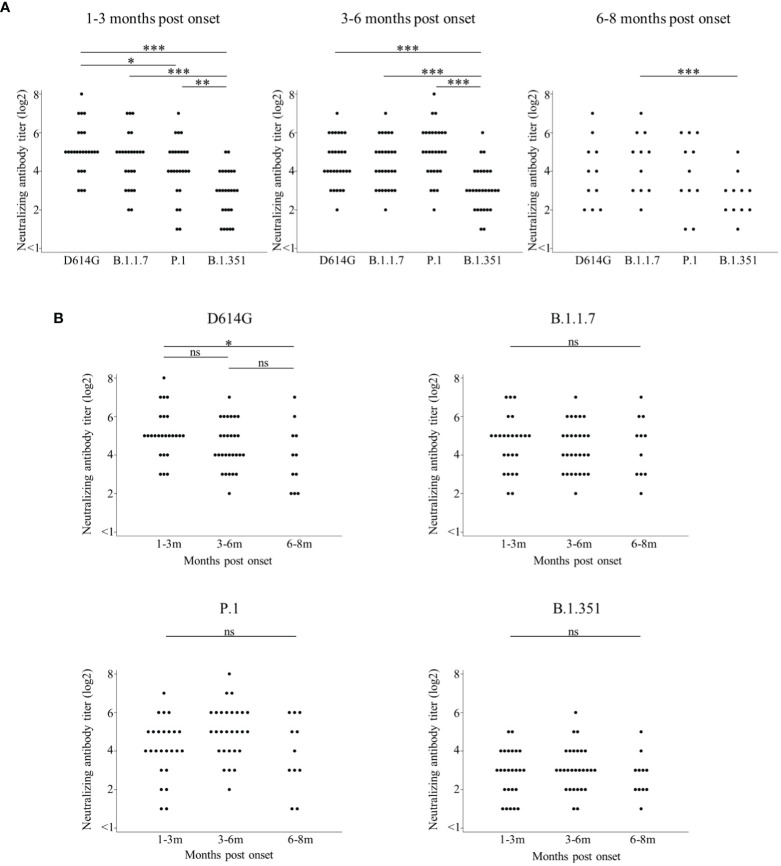
Comparisons of neutralizing antibody titers among D614G and three VOCs by the timing of sampling are shown in **(A)**. Longitudinal analysis of neutralizing antibody titer by D614G and three VOCs in **(B)**. Both figures focused on 28 patients with positive cross-Nabs for three VOCs. Vertical bars show the neutralizing antibody titer (log2) in **(A, B)**. Horizontal bars in **(A)** show the four variants (D614G mutation, B.1.1.7, P.1, and B.1.351) by the three groups of months post-onset (1-3m, 3-6m, and 6-8m). Horizontal bars in **(B)** show the three groups of months post-onset (1-3m, 3-6m, and 6-8m) for each of the four variants (D614G mutation, B.1.1.7, P.1, and B.1.351). Friedman’s test and Benjamini–Hochberg correction were performed to analyze both data. The level of statistical significance was set at *p* < 0.05. (*p < 0.05; **p < 0.01; ***p < 0.001; and ns, not significant).

Then, we focused on 28 patients with positive cross-Nabs for three VOCs and compared the Nab titer among the four variants by the timing of sampling (**Figure 3A**). These raw data were shown in **Supplementary Table 2**. The median Nab titer (log2) with IQR against D614G, B.1.1.7, P.1, and B.1.351 at 1-3 months post onset were 5 (5–6), 5 (4–5), 4 (4–5), and 3 (2–4), respectively. The Nab titer against B.1.351 was significantly lower than that against the other three variants, and the Nab titer against P.1 was significantly lower than that against D614G. The median Nab titer (log2) with IQR against D614G, B.1.1.7, P.1, and B.1.351 at 3-6 months post onset were 4 (4-5.5), 4 (3–5), 5 (4–6), and 3 (2–4), respectively. The Nab titer against B.1.351 was also significantly lower than that against the other three variants at 3-6 months post onset. The median Nab titer (log2) with IQR against D614G, B.1.1.7, P.1, and B.1.351 at 6-8 months post onset were 4 (2–5), 5 (3–6), 4 (3–6), and 3 (2–3), respectively. The Nab titer against B.1.351 was significantly lower than that against B.1.1.7.

Finally, we analyzed the retention of neutralizing activities over time against the four variants (**Figure 3B**) by rearranging the same data shown in **Figure 3A**. The Nab titer against D614G significantly decreased at 6-8 months post onset compared to 1-3 months post onset. Interestingly, each Nab titer against the three VOCs did not significantly change among three time points.

## Discussion

The purpose of this study was to examine the longevity of Nab activity of COVID-19 convalescent sera against D614G, and their neutralizing breadth against B.1.1.7, P.1, and B.1.351. We performed live virus neutralization assays against D614G and three VOCs to assess the Nab titers of COVID-19 convalescent sera. Infection of SARS-CoV-2 to the convalescent patients were confirmed from March 2020 to January 2021. Although B.1.1.7, P.1, and B.1.351 had already detected in Japan at the end of December 2020, only B.1.1.7 had spread in Japan and it rapidly increased from February 2021 (34, 35). A local surveillance in Japan also suggested that the B.1.1.7 became dominant after mid-April (36). These observations suggested that all participants in this study were likely to be infected with D614G. However, we could not completely exclude the possibility of VOCs infection due to the loss of the information about the viral sequence from patients’ nasopharyngeal specimens. In Addition, the imbalance of samples and small sample size were also our limitation.

This study revealed that all sera of ‘patients with pneumonia’ maintained cross-neutralizing activity against B.1.1.7, P.1, and B.1.351 until 3-8 months post infection. On the other hand, 14 out of 19 sera of ‘patients without pneumonia’ could not get or keep cross-neutralizing activity against three VOCs (**Figures 1**, **2A, B**). This reason might be that weak immune-response against SARS-CoV-2 lead to the low Nab titers of ‘patients without pneumonia’ against D614G compared to those of ‘patients with pneumonia’ (21, 37–40). The Nab titer against B.1.351 was lower than that against the other variants (**Figure 3A**), as similarly reported by other studies (14, 28, 41). The neutralizing titer against D614G significantly decreased in sera of 6-8 months post onset compared to those of 1-3 months post onset (**Figure 3B**). Several studies have reported that the peak neutralizing antibody titer is three to five weeks post onset, and decreases rapidly, then is sustained at low level for several months (42–44). The rapid early decay was shown to be caused by the short half-life of serum antibodies and by the short life of antibody-secreting cells; the maintenance of neutralizing antibody titers was supported by long-lived plasma cells to produce long-term antibodies (45). Further follow-up will be needed to confirm whether the specific Nab titer against D614G is maintained or not.

Interestingly and surprisingly, Nab titers against the three VOCs did not decrease until 6-8 months post onset (**Figure 3B**), possibly indicating that Nabs that recognize common epitopes were produced after infection, were selected for survival and sustained for a long time, while Nabs that recognized specific epitopes for a variant were produced in greater numbers after infection and decreased rapidly. Our results may reflect the increasing neutralizing breadth of antibodies which recognize common epitopes among VOCs (46–48). Importantly, SARS-CoV-2 RNA and proteins were detected in intestinal enterocytes several months post onset (46). These persisting viral antigens may stimulate memory B cells continuously. Then, B cells that produce neutralizing antibodies targeting the common epitope among variants can undergo further maturation in germinal center and as a result, produce high-affinity antibodies several months later. B cells that produce neutralizing antibodies targeting the common epitope among variants can undergo further maturation in germinal center and as a result, produce high-affinity antibodies several months later. Simultaneously, low-affinity antibodies disappear over time (48). Similar to our study, other study also showed that sera from convalescent patients infected with the ancestral SARS-CoV-2 maintained the cross-neutralizing antibody titers against B.1.1.7, P.1 and B.1.351 variants (49). Additionally, they compared cross-neutralizing activity in sera from convalescent patients infected with the ancestral strain, B.1.1.7, B.1.351 or B.1.617.2 SARS-CoV-2 variants. It was shown that the sera of convalescent patients infected with the ancestral strain maintained higher level of cross-neutralizing antibody titers than those infected with the other VOCs. The increasing neutralizing potency and breadth will help us develop effective hyper immunoglobulin and monoclonal variant-resistant antibodies like sotrovimab, which targets the surface of the RBD not overlapping with the ACE2 binding site and neutralizes B.1.1.7, P.1, B.1.351, and other variants (2). Neutralizing antibodies that recognize a common epitope for variants may be kept for a long time. SARS-CoV-2 specific functional CD 4^+^ T cell has also an important role to help the long lived S-specific B cell to produce high-affinity antibodies (50, 51). Recent study showed that mild COVID-19 patients induced fewer but functionally superior B cell than critical patients with mechanical ventilation (52). The diversity of B cell might be brought by CD4^+^ T cell. Further follow-up would be required to clarify the mechanism to get the long-lived immunity and cross-neutralizing activity against VOCs.

We should be careful in interpreting the meaning of ND. It remains unclear how much Nab titer determined by our method is required to protect reinfection. In this study, we did not examine whether fragment crystallizable (Fc) portions worked to recruit immune cells or serum complement as effectors. Some studies have shown that the Fc-mediated effector function of neutralizing antibodies against SARS-CoV-2 was essential for optimal therapy (53, 54). Therefore, the recoverees with low titer or ND in our study may still be protected against reinfection or severe disease after infection. Furthermore, we need to evaluate not only humoral immunity but also cell-mediated immunity. Cell-mediated immunity might be obtained because few mutations in the T-cell epitope of VOCs are known (41, 55), although L452R mutation, which is present in some variants (B.1.167 and B.1.427/429), escapes from HLA-24 cell-mediated immunity (56). Therefore, even if the neutralization activity falls below the detection limit in the long term, convalescent COVID-19 patients might be protected from VOCs.

## Data Availability Statement

The original contributions presented in the study are included in the article/**Supplementary Material**. Further inquiries can be directed to the corresponding author.

## Ethics Statement

The studies involving human participants were reviewed and approved by The ethics committee of Kobe University Graduate School of Medicine (ID: B200200) and Hyogo Prefectural Kakogawa Medical Center. The patients/participants provided their written informed consent to participate in this study.

## Author Contributions

All authors contributed to the concept of this article. YK drafted the manuscript. MN and YM provided revisions. YK, JA, MN, and YM analyzed the data. YK, SSu, KF, and LT did the experiments. YM and YN supervised the experiments. SI, YT, SSa, SN, TK, MY, and TN collected the samples. YM conducted the project. All authors approved the final version of the manuscript.

## Funding

This work was supported by Hyogo Prefectural Government and the Kansai Economic Federation (KANKEIREN). The funders had no role in this study.

## Conflict of Interest

The authors declare that the research was conducted in the absence of any commercial or financial relationships that could be construed as a potential conflict of interest.

## Publisher’s Note

All claims expressed in this article are solely those of the authors and do not necessarily represent those of their affiliated organizations, or those of the publisher, the editors and the reviewers. Any product that may be evaluated in this article, or claim that may be made by its manufacturer, is not guaranteed or endorsed by the publisher.

## References

[1] Worldmeter. COVID-19 Coronavirus Pandemic (2021). Available at: https://www.worldometers.info/coronavirus/.

[2] Corti D Purcell LA Snell G Veesler D. Tackling COVID-19 With Neutralizing Monoclonal Antibodies. Cell (2021) 184(12):3086–108. doi: 10.1016/j.cell.2021.05.005 PMC8152891 34087172

[3] Pormohammad A Zarei M Ghorbani S Mohammadi M Razizadeh MH Turner DL. Efficacy and Safety of COVID-19 Vaccines: A Systematic Review and Meta-Analysis of Randomized Clinical Trials. Vaccines (Basel) (2021) 9(5):467. doi: 10.3390/vaccines9050467 34066475 PMC8148145

[4] Mair-Jenkins J Saavedra-Campos M Baillie JK Cleary P Khaw FM Lim WS. The Effectiveness of Convalescent Plasma and Hyperimmune Immunoglobulin for the Treatment of Severe Acute Respiratory Infections of Viral Etiology: A Systematic Review and Exploratory Meta-Analysis. J Infect Dis (2015) 211(1):80–90. doi: 10.1093/infdis/jiu396 25030060 PMC4264590

[5] van Griensven J De Weiggheleire A Delamou A Smith PG Edwards T Vandekerckhove P. The Use of Ebola Convalescent Plasma to Treat Ebola Virus Disease in Resource-Constrained Settings: A Perspective From the Field. Clin Infect Dis (2016) 62(1):69–74. doi: 10.1093/cid/civ680 26261205 PMC4678103

[6] Piechotta V Iannizzi C Chai KL Valk SJ Kimber C Dorando E. Convalescent Plasma or Hyperimmune Immunoglobulin for People With COVID-19: A Living Systematic Review. Cochrane Database Syst Rev (2021) 5:CD013600. doi: 10.1002/14651858.CD013600.pub4 34013969 PMC8135693

[7] Bansal V Mahapure KS Mehra I Bhurwal A Tekin A Singh R. Mortality Benefit of Convalescent Plasma in COVID-19: A Systematic Review and Meta-Analysis. Front Med (Lausanne) (2021) 8:624924. doi: 10.3389/fmed.2021.624924 33898477 PMC8062901

[8] Lu RM Hwang YC Liu IJ Lee CC Tsai HZ Li HJ. Development of Therapeutic Antibodies for the Treatment of Diseases. J BioMed Sci (2020) 27(1):1. doi: 10.1186/s12929-019-0592-z 31894001 PMC6939334

[9] Mullard A. FDA Approves Antibody Cocktail for Ebola Virus. Nat Rev Drug Discov (2020) 19(12):827. doi: 10.1038/d41573-020-00197-8 33144717

[10] National Institutes of Health. Anti-SARS-CoV-2 Monoclonal Antibodies (2021). Available at: https://www.covid19treatmentguidelines.nih.gov/therapies/anti-sars-cov-2-antibody-products/anti-sars-cov-2-monoclonal-antibodies/.

[11] Chugai Pharmaceutical Co., LTD. The Antibody Cocktail, RONAPREVE for Intravenous Infusion Set Receives the World’s First Regulatory Approval From MHLW for COVID-19 (2021). Available at: https://www.chugai-pharm.co.jp/english/news/detail/20210719220000_843.html.

[12] Callaway E. The Coronavirus is Mutating - Does it Matter? Nature (2020) 585(7824):174–7. doi: 10.1038/d41586-020-02544-6 32901123

[13] World Health Organization. Tracking SARS-CoV-2 Variants (2021). Available at: https://www.who.int/en/activities/tracking-SARS-CoV-2-variants/.

[14] Dejnirattisai W Zhou D Supasa P Liu C Mentzer AJ Ginn HM. Antibody Evasion by the P.1 Strain of SARS-CoV-2. Cell (2021) 184(11):2939–54.e9. doi: 10.1016/j.cell.2021.03.055 33852911 PMC8008340

[15] Li Q Wu J Nie J Zhang L Hao H Liu S. The Impact of Mutations in SARS-CoV-2 Spike on Viral Infectivity and Antigenicity. Cell (2020) 182(5):1284–94.e9. doi: 10.1016/j.cell.2020.07.012 32730807 PMC7366990

[16] Johnson BA Xie X Kalveram B Lokugamage KG Muruato A Zou J. Furin Cleavage Site Is Key to SARS-CoV-2 Pathogenesis. bioRxiv (2020) 2020.08.26.268854. doi: 10.1101/2020.08.26.268854

[17] Kumar S Thambiraja TS Karuppanan K Subramaniam G. Omicron and Delta Variant of SARS-CoV-2: A Comparative Computational Study of Spike Protein. J Med Virol (2021) 1–9. doi: 10.1002/jmv.27526 34914115

[18] Davies NG Abbott S Barnard RC Jarvis CI Kucharski AJ Munday JD. Estimated Transmissibility and Impact of SARS-CoV-2 Lineage B.1.1.7 in England. Science (2021) 372(6538):eabg3055. doi: 10.1126/science.abg3055 33658326 PMC8128288

[19] Greaney AJ Starr TN Gilchuk P Zost SJ Binshtein E Loes AN. Complete Mapping of Mutations to the SARS-CoV-2 Spike Receptor-Binding Domain That Escape Antibody Recognition. Cell Host Microbe (2021) 29(1):44–57.e9. doi: 10.1016/j.chom.2020.11.007 33259788 PMC7676316

[20] Nelson G Buzko O Spilman P Niazi K Rabizadeh S Soon-Shiong P. Molecular Dynamic Simulation Reveals E484K Mutation Enhances Spike RBD-ACE2 Affinity and the Combination of E484K, K417N and N501Y Mutations (501Y.V2 Variant) Induces Conformational Change Greater Than N501Y Mutant Alone, Potentially Resulting in an Escape Mutant. bioRxiv (2021) 2021.01.13.426558. doi: 10.1101/2021.01.13.426558

[21] Caniels TG Bontjer I van der Straten K Poniman M Burger JA Appelman B. Emerging SARS-CoV-2 Variants of Concern Evade Humoral Immune Responses From Infection and Vaccination. medRxiv (2021) 2021.05.26.21257441. doi: 10.1101/2021.05.26.21257441 PMC8442901 34516917

[22] Wang Y Zhang L Li Q Liang Z Li T Liu S. The Significant Immune Escape of Pseudotyped SARS-CoV-2 Variant Omicron. Emerg Microbes Infect (2022) 11(1):1–5. doi: 10.1080/22221751.2021.2017757 34890524 PMC8725892

[23] Dejnirattisai W Shaw RH Supasa P Liu C Stuart AS Pollard AJ. Reduced Neutralisation of SARS-CoV-2 Omicron B.1.1.529 Variant by Post-Immunisation Serum. Lancet (2022) 399(10321):234–6. doi: 10.1016/s0140-6736(21)02844-0 PMC8687667 34942101

[24] Tokumasu R Weeraratne D Snowdon J Parida L Kudo M Koyama T. Introductions and Evolutions of SARS-CoV-2 Strains in Japan. medRxiv (2021) 2021.02.26.21252555. doi: 10.1101/2021.02.26.21252555

[25] National Institute of Infectious Diseases. Current Situation of Infection, April 27, 2021 (2021). Available at: https://www.niid.go.jp/niid/en/2019-ncov-e/10345-covid19-ab32th-en.html.

[26] National Institute of Infectious Diseases. Current Situation of Infection, August 18, 2021 (2021). Available at: https://www.niid.go.jp/niid/en/2019-ncov-e/10598-covid19-ab48th-en.html.

[27] National Institute of Infectious Diseases. Current Situation of Infection (2021). Available at: https://www.niid.go.jp/niid/en/2019-ncov-e/10829-covid19-ab62th-en.html.

[28] Betton M Livrozet M Planas D Fayol A Monel B Védie B. Sera Neutralizing Activities Against SARS-CoV-2 and Multiple Variants Six Month After Hospitalization for COVID-19. Clin Infect Dis (2021) 73(6):e1337–44. doi: 10.1093/cid/ciab308 PMC8083257 33851216

[29] Terpos E Stellas D Rosati M Sergentanis TN Hu X Politou M. SARS-CoV-2 Antibody Kinetics Eight Months From COVID-19 Onset: Persistence of Spike Antibodies But Loss of Neutralizing Antibodies in 24% of Convalescent Plasma Donors. Eur J Intern Med (2021) 89:87–96. doi: 10.1016/j.ejim.2021.05.010 34053848 PMC8128693

[30] Sonnleitner ST Prelog M Jansen B Rodgarkia-Dara C Gietl S Schonegger CM. Maintenance of Neutralizing Antibodies Over Ten Months in Convalescent SARS-CoV-2 Afflicted Patients. Transbound Emerg Dis (2021) 1–10. doi: 10.1111/tbed.14130 PMC8242897 33960696

[31] Edara VV Hudson WH Xie X Ahmed R Suthar MS. Neutralizing Antibodies Against SARS-CoV-2 Variants After Infection and Vaccination. JAMA (2021) 325(18):1896–8. doi: 10.1001/jama.2021.4388 PMC7980146 33739374

[32] Tjan LH Furukawa K Nagano T Kiriu T Nishimura M Arii J. Early Differences in Cytokine Production by Severity of Coronavirus Disease 2019. J Infect Dis (2021) 223(7):1145–9. doi: 10.1093/infdis/jiab005 PMC7928883 33411935

[33] Furukawa K Arii J Nishimura M Tjan LH Lystia Poetranto A Ren Z. Seroepidemiological Survey of the Antibody for Severe Acute Respiratory Syndrome Coronavirus 2 With Neutralizing Activity at Hospitals: A Cross-Sectional Study in Hyogo Prefecture, Japan. JMA J (2021) 4(1):41–9. doi: 10.31662/jmaj.2020-0094 PMC7872787 33575502

[34] Latif AA Mullen JL Alkuzweny M Tsueng G Cano M Haag E. Japan Mutation Report: Outbreak.Info (2021). Available at: https://outbreak.info/location-reports?loc=JPN&selected=Alpha.

[35] Latif AA Mullen JL Alkuzweny M Tsueng G Cano M Haag E. B.1.1.7 Lineage Report: Outbreak.Info (2021). Available at: https://outbreak.info/situation-reports?loc=JPN&pango=B.1.1.7&selected=JPN.

[36] Hirotsu Y Omata M. SARS-CoV-2 B.1.1.7 Lineage Rapidly Spreads and Replaces R.1 Lineage in Japan: Serial and Stationary Observation in a Community. Infect Genet Evol (2021) 95:105088. doi: 10.1016/j.meegid.2021.105088 34560289 PMC8454025

[37] Tjan LH Nagano T Furukawa K Nishimura M Arii J Fujinaka S. The Neutralizing Antibody Response Against Severe Acute Respiratory Syndrome Coronavirus 2 and the Cytokine/Chemokine Release in Patients With Different Levels of Coronavirus Diseases 2019 Severity: Cytokine Storm Still Persists Despite Viral Disappearance in Critical Patients. JMA J (2021) 4(1):1–7. doi: 10.31662/jmaj.2020-0083 33575497 PMC7872782

[38] Savage HR Santos VS Edwards T Giorgi E Krishna S Planche TD. Prevalence of Neutralising Antibodies Against SARS-CoV-2 in Acute Infection and Convalescence: A Systematic Review and Meta-Analysis. PloS Negl Trop Dis (2021) 15(7):e0009551. doi: 10.1371/journal.pntd.0009551 34237072 PMC8291969

[39] Pradenas E Trinite B Urrea V Marfil S Avila-Nieto C Rodriguez de la Concepcion ML. Stable Neutralizing Antibody Levels 6 Months After Mild and Severe COVID-19 Episodes. Med (NY) (2021) 2(3):313–20.e4. doi: 10.1016/j.medj.2021.01.005 PMC7847406 33554155

[40] Yue S Li Z Lin Y Yang Y Yuan M Pan Z. Sensitivity of SARS-CoV-2 Variants to Neutralization by Convalescent Sera and a VH3-30 Monoclonal Antibody. Front Immunol (2021) 12:751584. doi: 10.3389/fimmu.2021.751584 34630430 PMC8495157

[41] Geers D Shamier MC Bogers S den Hartog G Gommers L Nieuwkoop NN. SARS-CoV-2 Variants of Concern Partially Escape Humoral But Not T-Cell Responses in COVID-19 Convalescent Donors and Vaccinees. Sci Immunol (2021) 6(59):eabj1750. doi: 10.1126/sciimmunol.abj1750 34035118 PMC9268159

[42] Röltgen K Powell AE Wirz OF Stevens BA Hogan CA Najeeb J. Defining the Features and Duration of Antibody Responses to SARS-CoV-2 Infection Associated With Disease Severity and Outcome. Sci Immunol (2020) 5(54):eabe0240. doi: 10.1126/sciimmunol.abe0240 33288645 PMC7857392

[43] Ibarrondo FJ Fulcher JA Goodman-Meza D Elliott J Hofmann C Hausner MA. Rapid Decay of Anti-SARS-CoV-2 Antibodies in Persons With Mild Covid-19. N Engl J Med (2020) 383(11):1085–7. doi: 10.1056/NEJMc2025179 PMC7397184 32706954

[44] Wu J Liang B Chen C Wang H Fang Y Shen S. SARS-CoV-2 Infection Induces Sustained Humoral Immune Responses in Convalescent Patients Following Symptomatic COVID-19. Nat Commun (2021) 12(1):1813. doi: 10.1038/s41467-021-22034-1 33753738 PMC7985370

[45] Hammarlund E Thomas A Amanna IJ Holden LA Slayden OD Park B. Plasma Cell Survival in the Absence of B Cell Memory. Nat Commun (2017) 8(1):1781. doi: 10.1038/s41467-017-01901-w 29176567 PMC5701209

[46] Gaebler C Wang Z Lorenzi JCC Muecksch F Finkin S Tokuyama M. Evolution of Antibody Immunity to SARS-CoV-2. Nature (2021) 591(7851):639–44. doi: 10.1038/s41586-021-03207-w PMC8221082 33461210

[47] Wang Z Muecksch F Schaefer-Babajew D Finkin S Viant C Gaebler C. Naturally Enhanced Neutralizing Breadth Against SARS-CoV-2 One Year After Infection. Nature (2021) 595(7867):426–31. doi: 10.1038/s41586-021-03696-9 PMC8277577 34126625

[48] Moriyama S Adachi Y Sato T Tonouchi K Sun L Fukushi S. Temporal Maturation of Neutralizing Antibodies in COVID-19 Convalescent Individuals Improves Potency and Breadth to Circulating SARS-CoV-2 Variants. Immunity (2021) 54(8):1841–52.e4. doi: 10.1016/j.immuni.2021.06.015 34246326 PMC8249673

[49] Dupont L Snell LB Graham C Seow J Merrick B Lechmere T. Neutralizing Antibody Activity in Convalescent Sera From Infection in Humans With SARS-CoV-2 and Variants of Concern. Nat Microbiol (2021) 6(11):1433–42. doi: 10.1038/s41564-021-00974-0 PMC8556155 34654917

[50] Le Bert N Tan AT Kunasegaran K Tham CYL Hafezi M Chia A. SARS-CoV-2-Specific T Cell Immunity in Cases of COVID-19 and SARS, and Uninfected Controls. Nature (2020) 584(7821):457–62. doi: 10.1038/s41586-020-2550-z 32668444

[51] Grifoni A Weiskopf D Ramirez SI Mateus J Dan JM Moderbacher CR. Targets of T Cell Responses to SARS-CoV-2 Coronavirus in Humans With COVID-19 Disease and Unexposed Individuals. Cell (2020) 181(7):1489–501.e15. doi: 10.1016/j.cell.2020.05.015 32473127 PMC7237901

[52] Pusnik J Richter E Schulte B Dolscheid-Pommerich R Bode C Putensen C. Memory B Cells Targeting SARS-CoV-2 Spike Protein and Their Dependence on CD4(+) T Cell Help. Cell Rep (2021) 35(13):109320. doi: 10.1016/j.celrep.2021.109320 34146478 PMC8192958

[53] Winkler ES Gilchuk P Yu J Bailey AL Chen RE Chong Z. Human Neutralizing Antibodies Against SARS-CoV-2 Require Intact Fc Effector Functions for Optimal Therapeutic Protection. Cell (2021) 184(7):1804–20.e16. doi: 10.1016/j.cell.2021.02.026 33691139 PMC7879018

[54] Chan CEZ Seah SGK Chye H Massey S Torres M Lim APC. The Fc-Mediated Effector Functions of a Potent SARS-CoV-2 Neutralizing Antibody, SC31, Isolated From an Early Convalescent COVID-19 Patient, are Essential for the Optimal Therapeutic Efficacy of the Antibody. PloS One (2021) 16(6):e0253487. doi: 10.1371/journal.pone.0253487 34161386 PMC8221499

[55] Grifoni A Sidney J Vita R Peters B Crotty S Weiskopf D. SARS-CoV-2 Human T Cell Epitopes: Adaptive Immune Response Against COVID-19. Cell Host Microbe (2021) 29(7):1076–92. doi: 10.1016/j.chom.2021.05.010 PMC8139264 34237248

[56] Motozono C Toyoda M Zahradnik J Saito A Nasser H Tan TS. SARS-CoV-2 Spike L452R Variant Evades Cellular Immunity and Increases Infectivity. Cell Host Microbe (2021) 29(7):1124–36.e11. doi: 10.1016/j.chom.2021.06.006 34171266 PMC8205251

