# Supplementary material for: Cross-Neutralizing Breadth and Longevity Against SARS-CoV-2 Variants After Infections
Source: Front Immunol. 2022 Feb 24;13:773652. doi: 10.3389/fimmu.2022.773652 (PMC8907139; doi:10.3389/fimmu.2022.773652)
Supplement: Supplementary file 1 [file DataSheet_1.pdf]

Supplementary Table 1

Patient ID	Severity	Months post onset	Neutralizing antibody titer (log2)			
			D614G	B.1.1.7	P.1	B.1.351
1	asymptomatic	1-3	4	2	2	1
		3-6	2	2	2	<1
		6-8	No sample			
2	asymptomatic	1-3	No sample			
		3-6	3	3	2	<1
		6-8	1	2	3	<1
3	asymptomatic	1-3	4	2	1	1
		3-6	2	1	1	<1
		6-8	No sample			
4	asymptomatic	1-3	3	2	1	<1
		3-6	1	<1	<1	<1
		6-8	No sample			
5	mild	1-3	3	2	1	<1
		3-6	2	2	1	<1
		6-8	2	2	<1	<1
6	mild	1-3	5	5	5	4
		3-6	4	4	5	3
		6-8	3	3	3	3
7	mild	1-3	4	3	4	1
		3-6	3	2	2	1
		6-8	2	2	1	1
8	mild	1-3	2	<1	<1	<1
		3-6	2	<1	<1	<1
		6-8	<1	<1	<1	<1
9	mild	1-3	5	4	2	2
		3-6	2	5	3	2
		6-8	2	3	1	2

10	mild	1-3	No sample			
		3-6	3	3	4	3
		6-8	3	3	3	2
11	mild	1-3	1	1	<1	<1
		3-6	2	1	2	<1
		6-8	No sample			
12	mild	1-3	3	2	2	2
		3-6	2	2	1	<1
		6-8	No sample			
13	mild	1-3	3	3	5	2
		3-6	4	3	5	2
		6-8	No sample			
14	mild	1-3	3	2	1	<1
		3-6	2	1	<1	<1
		6-8	No sample			
15	mild	1-3	4	3	3	2
		3-6	3	2	2	<1
		6-8	No sample			
16	mild	1-3	4	3	3	2
		3-6	2	1	<1	<1
		6-8	No sample			
17	mild	1-3	2	2	2	<1
		3-6	1	3	2	<1
		6-8	1	1	2	<1
18	mild	1-3	1	<1	<1	<1
		3-6	<1	<1	<1	<1
		6-8	<1	<1	<1	<1
19	mild	1-3	3	5	4	1
		3-6	5	4	2	1
		6-8	3	3	3	<1
20	moderate	1-3	5	5	4	4
		3-6	6	6	6	3
		6-8	6	7	6	4

21	moderate	1-3	3	2	2	1
		3-6	3	4	5	2
		6-8	2	4	3	2
22	moderate	1-3	5	4	3	3
		3-6	5	4	6	2
		6-8	No sample			
23	moderate	1-3	5	5	4	1
		3-6	5	3	5	1
		6-8	No sample			
24	moderate	1-3	4	5	3	2
		3-6	4	3	4	4
		6-8	No sample			
25	severe	1-3	No sample			
		3-6	6	5	5	2
		6-8	5	6	4	3
26	severe	1-3	6	7	6	4
		3-6	4	5	6	4
		6-8	No sample			
27	severe	1-3	5	5	4	3
		3-6	5	4	4	3
		6-8	4	5	5	3
28	severe	1-3	5	6	5	4
		3-6	5	5	6	3
		6-8	4	5	6	2
29	severe	1-3	5	7	4	3
		3-6	4	5	5	3
		6-8	5	5	5	3
30	severe	1-3	6	5	5	3
		3-6	4	4	6	3
		6-8	No sample			
31	severe	1-3	5	5	6	3
		3-6	6	6	8	4
		6-8	No sample			

32	severe	1-3	5	6	4	4
		3-6	7	5	5	4
		6-8	No sample			
33	severe	1-3	4	2	1	1
		3-6	3	3	3	3
		6-8	No sample			
34	severe	1-3	5	3	1	1
		3-6	4	4	3	2
		6-8	No sample			
35	severe	1-3	5	5	5	4
		3-6	5	6	6	4
		6-8	No sample			
36	severe	1-3	8	4	5	3
		3-6	6	4	5	3
		6-8	No sample			
37	severe	1-3	3	3	4	2
		3-6	6	6	7	5
		6-8	No sample			
38	severe	1-3	6	5	4	3
		3-6	3	3	6	4
		6-8	No sample			
39	severe	1-3	7	4	5	3
		3-6	4	3	4	3
		6-8	No sample			
40	critical	1-3	No sample			
		3-6	6	7	7	6
		6-8	7	6	6	5
41	critical	1-3	7	5	7	5
		3-6	4	6	6	5
		6-8	No sample			
42	critical	1-3	7	7	6	5
		3-6	5	5	4	3
		6-8	No sample			

Supplementary Table 2

Patient ID	Neutralizing antibody titer (log2)											
	1-3 month post onset				3-6 month post onset				6-8 month post onset			
	D614G	B.1.1.7	P.1	B.1.351	D614G	B.1.1.7	P.1	B.1.351	D614G	B.1.1.7	P.1	B.1.351
6	5	5	5	4	4	4	5	3	3	3	3	3
7	4	3	4	1	3	2	2	1	2	2	1	1
9	5	4	2	2	2	5	3	2	2	3	1	2
10	No sample				3	3	4	3	3	3	3	2
13	3	3	5	2	4	3	5	2	No sample			
20	5	4	4	4	6	6	6	3	6	7	6	4
21	3	2	2	1	3	4	5	2	2	4	3	2
22	5	4	3	3	5	4	6	2	No sample			
23	5	5	4	1	5	3	5	1	No sample			
24	4	5	3	2	4	3	4	4	No sample			
25	No sample				6	5	5	2	5	6	4	3
26	6	7	6	4	4	5	6	4	No sample			
27	5	5	4	3	5	4	4	3	4	5	5	3
28	5	6	5	4	5	5	6	3	4	5	6	2
29	5	7	4	3	4	5	5	3	5	5	5	3
30	6	5	5	3	4	4	6	3	No sample			
31	5	5	6	3	6	6	8	4	No sample			

32	5	6	4	4	7	5	5	4	No sample			
33	4	2	1	1	3	3	3	3	No sample			
34	5	3	1	1	4	4	3	2	No sample			
35	5	5	5	4	5	6	6	4	No sample			
36	8	4	5	3	6	4	5	3	No sample			
37	3	3	4	3	6	6	7	5	No sample			
38	6	5	4	2	3	3	6	4	No sample			
39	7	4	5	3	4	3	4	3	No sample			
40	No sample				6	7	7	6	7	6	6	5
41	7	5	7	3	4	6	6	5	No sample			
42	7	6	6	5	5	5	4	3	No sample			
